# Neuropil contraction in relation to *Complement C4 gene* copy numbers in independent cohorts of adolescent-onset and young adult-onset schizophrenia patients–a pilot study

**DOI:** 10.1038/s41398-018-0181-z

**Published:** 2018-07-19

**Authors:** Konasale M. Prasad, Kodavali V. Chowdari, Leonardo A. D’Aiuto, Satish Iyengar, Jeffrey A. Stanley, Vishwajit L. Nimgaonkar

**Affiliations:** 1 Department of Psychiatry, University of Pittsburgh School of Medicine, Pittsburgh, PA USA; 2 Department of Statistics, University of Pittsburgh, Pittsburgh, PA USA; 3 Department of Psychiatry and Behavioral Neuroscience, Wayne State University School of Medicine, Detroit, MI USA; 4 Department of Human Genetics, Graduate School of Public Health, University of Pittsburgh, Pittsburgh, PA USA

## Abstract

A recent report suggested *Complement 4 (C4A)* gene copy numbers (GCN) as risk factors for schizophrenia. Rodent model showed association of *C4* with synaptic pruning suggesting its pathophysiological significance (Sekar, A. et al. (2016)). We, therefore, predicted that *C4A* GCN would be positively correlated with neuropil contraction in the human brain among schizophrenia patients showing more prominent correlations in ventral regions among young adults and dorsal regions among adolescents since neuromaturation progresses dorsoventrally. Whole-brain, multi-voxel, in vivo phosphorus magnetic resonance spectroscopy (^31^P MRS) assessed neuropil changes by estimating levels of membrane phospholipid (MPL) precursors and catabolites. Increased MPL catabolites and/or decreased MPL precursors indexed neuropil contraction. Digital droplet PCR-based assay was used to estimate *C4A* and *C4B* GCN. We evaluated two independent cohorts (young adult-onset early-course schizophrenia (YASZ = 15) and adolescent-onset schizophrenia (AOSZ = 12) patients), and controls matched for each group, *n* = 22 and 15, respectively. Separate forward stepwise linear regression models with Akaike information Criterion were built for MPL catabolites and precursors. *YASZ cohort:* Consistent with the rodent model (Sekar, A. et al. 2016)), *C4A* GCN positively correlated with neuropil contraction (increased pruning/decreased formation) in the inferior frontal cortex and inferior parietal lobule. *AOSZ cohort: C4A* GCN positively correlated with neuropil contraction in the dorsolateral prefrontal cortex and thalamus. Exploratory analysis of *C4B* GCN showed positive correlation with neuropil contraction in the cerebellum and superior temporal gyrus among YASZ while AOSZ showed neuropil contraction in the prefrontal and subcortical structures. Thus, *C4A and C4B* GCN are associated with neuropil contraction in regions often associated with schizophrenia, and may be neuromaturationally dependent.

## Introduction

Schizophrenia is a severe brain disorder which costs over $155 billion a year in the United States^1^. Available treatments are symptomatic leading to poor long-term social outcome^2–5^. A better understanding of pathophysiology may help develop new treatments. One of the neurodevelopmental models that propose excessive loss of synapses^6^ may be one such mechanism. Convergent animal^7,8^, human developmental^9^, neuroimaging^10,11^, postmortem^12,13^, and computational modeling^14,15^ data suggest that increased neuropil loss predates^16–23^ and continues after the onset of psychosis^24,25^, and may predict short-term outcome^26^. The genetic underpinnings of excessive synaptic pruning are poorly understood in humans.

Recently, independent lines of evidence suggest that *Complement 4* (*C4A)* gene copy numbers (GCN) are associated with schizophrenia risk and synaptic pruning. This is important because a number of prior studies reported altered peripheral blood complement protein levels in schizophrenia but the results were inconsistent^27–36^ and the pathophysiological significance of such alterations was unclear since the peripheral complement proteins may not cross blood brain barrier^37^.

Persuasive results from a study by Sekar et al. (2016)^38^ demonstrated that a copy number variant (CNV) accounts for a portion of the risk in Human Leukocyte Antigen (HLA) region reported repeatedly in genetic association studies of schizophrenia^39–42^. This CNV consists of ‘cassettes’, denoted by ‘R-C-C-X’ that comprises *STK19* (***R****P1*), ***C****4 (C4A or C4B)*, ***C****YP21A1* or *CYP21A2*, and *TN****X****B*^43^. The C4 sequences can encode *C4A* or *C4B*, which are isotypes of *C4* with > 99% sequence homology; however, the translational products differ in antigen affinities and hemolytic activity^44–46^. A recombination site at *CYP21A2* leads to mono-, bi-, and tri-modular RCCX cassettes (and rarely, 4 modules) that can generate multiple functional copies of *C4A*/*C4B*, while retaining just one functional copy of the remaining genes^47^. Thus, each chromosome commonly has 1–3 functional copies of *C4A*/*C4B*, (rarely, 0 or 4 copies), for a typical total of 0–6 copies/individual; further, transcription of *C4A*/*C4B* can be impacted by an intronic human endogenous retroviral (HERV) sequence^48^. Sekar et al.^38^ found that *C4A*, but not *C4B* GCN are associated with higher risk for schizophrenia. In post-mortem brain samples of schizophrenia patients, the expression of *C4A* and *C4B* genes was proportional to the number of *C4* GCN with higher expression in 5 brain regions (namely the frontal cortex, cingulate cortex, parietal cortex, cerebellum, corpus callosum and orbitofrontal cortex) by approximately 40% among schizophrenia patients compared to controls^38^. A rodent model showed decreased synaptic pruning in *C4*-deficient mice. The RCCX CNV is also associated with risk for auto-immune disorders that have altered prevalence among schizophrenia patients^44,49,50^. Recently, *C4* mRNA levels in plasma have been correlated with severity of psychopathology in schizophrenia^51^. Another study found correlations between predicted *C4A* transcription and impairment in memory^52^. Complement proteins were also associated with risk for schizophrenia^53^, and with thinning of superior frontal cortex^54^. Thus, in humans, the complement system serves diverse immune and neural functions^55,37^ and suggest that abnormal *C4A* function contributes to schizophrenia pathogenesis.

We examined the relationship of *C4* GCN with neuropil contraction/expansion in a human context within two independent cohorts of schizophrenia patients and healthy controls (HC). We examined neuropil because direct examination of synapses in live human subjects is challenging as the synapses are embedded in the neuropil. Neuropil is a synaptically dense region composed of dendrites, unmyelinated axons and glial filaments with relatively few cell bodies^56,57^. Phosphorus magnetic resonance spectroscopy (^31^P MRS) is used to assess changes in neuronal membrane expansion/contraction within the neuropil by estimating the availability of membrane phospholipid (MPL) precursors (phosphocholine, PC; phosphoethanolamine, PE) and catabolites (glycerophosphocholine, GPC; glycerophosphoethanolamine, GPE). PC, PE, GPC, and GPE can be reliably measured in the brain by ^31^P MRS^58^. Specificity of these measurements to neuropil compared to gray matter measurements is supported by convergent data from animal lesion^59^, cellular model^60^, human postmortem^61^ and neurodevelopmental studies^9,62^. Greater sensitivity for age-related changes of MPL metabolites to neuropil changes compared to gray matter metrics is provided by human developmental studies^9,63^. Thus, ^31^P MRS that assesses molecular biochemistry of neuropil is superior to gray matter measures that represent composite physical measurement of total volume of interneurons, synapses, axonal terminals, dendritic arborization, neuronal soma, glia, microvasculature and interneuronal space^64,65^.

The rationale for the superiority of ^31^P MRS has been described in prior publications^9,11,62^. Briefly, synapse/dendritic spine formation and dendritic branching requires expansion of dendritic/axonal membranes. This expansion requires increased MPL synthesis, with resultant increase in MPL precursor levels (PC + PE). This is supported by elevation of PC + PE at the time and site of neuropil growth spurts^59,62^. Likewise, neuropil contraction or pruning are associated with breakdown of MPLs leading to elevated GPC + GPE, which is, also, noted at the time and site of synaptic pruning^59,66^. Since expansion and contraction of neuronal membranes largely occurs at the dendrites and axonal endings during neuropil growth/contraction with a considerably smaller contribution from changes in neuronal soma size, glial expansion and myelin content^67–69^, changes in MPL metabolites are thought to more specifically and sensitively index changes in axonal endings, dendritic branches and synapses.

We examined two independent cohorts–young adult-onset schizophrenia (YASZ) cohort (*n* = 15) and adolescent-onset schizophrenia (AOSZ) patients (*n* = 12)- and two HC cohorts age-matched for each group (*n* = 22 and 15, respectively). Examination of two cohorts helps replicate the findings and explore the association of *C4A* repeats with neuropil contraction in a developmental context. We selected MRS voxels from five of the six brain regions that showed increased *C4A* and *C4B* expression in postmortem tissue^38^. These included the frontal cortex (the dorsolateral prefrontal cortex (DLPFC), inferior frontal cortex (IFC), ventral PFC (VPFC)), cingulate cortex (anterior cingulate cortex (ACC) and posterior cingulate cortex (PCC)), parietal lobe (inferior parietal lobule (IPL), superior parietal lobule (SPL)), orbitofrontal cortex (OFC) and cerebellum but not corpus callosum because of lower signal-to-noise ratio (SNR) of ^31^P MRS data for this region. Since prior studies showed progression of brain maturation from the dorsal (e.g., temporal-parietal regions) to the ventral (e.g., prefrontal cortices) regions involving elimination of overproduced synapses that is prominent from late childhood to the third decade of life^70–75^, we selected the superior temporal gyrus (STG) in addition to the above regions. Subcortical structures (thalamus, caudate, and ventral and dorsal hippocampus) were, also, included because these structures show continued maturation from childhood to late adulthood^76–78^. Thus, fourteen regions were selected on both hemispheres so that we could examine neuropil alterations in regions that showed increased *C4* expression by Sekar et al., and neurodevelopmental changes in the dorsal (STG, PCC, IPL, SPL), ventral (DLPFC, ACC, OFC, IFC, VPFC) and subcortical regions.

Our primary hypothesis was that the GPC + GPE levels would be elevated with increasing *C4A* repeats in the frontal (DLFPC, IFC, VPFC), cingulate (ACC, PCC), parietal (IPL, SPL), the OFC and the cerebellum in both cohorts. We, further, hypothesized that the GPC + GPE levels would be elevated with increasing *C4A* repeats in YASZ cohort in the ventral brain regions (DLFPC, IFC, OFC, ACC, and VPFC) whereas the AOSZ would show such elevations in the dorsal regions (STG, PCC, IPL, and SPL). We predicted increased GPC + GPE in the hippocampus, caudate and thalamus in both cohorts because of protracted maturation. We investigated whether the *C4A* GCN would be associated with decreased PC + PE (MPL precursors) levels suggesting decreased neuropil expansion, and explored whether *C4B* GCN would be similarly correlated with GPC + GPE and PC + PE levels in these regions similar to *C4A* associations.

## Methods

### SAMPLE 1: YASZ and age-matched HC

#### Clinical evaluations

YASZ between 18–44 years of age with DSM-IV schizophrenia/schizoaffective disorder and ≤ 5 years of illness from the onset of psychotic symptoms were eligible to be enrolled at the University of Pittsburgh Medical Center. The diagnosis was confirmed in a consensus meeting of experienced diagnosticians^79^ after reviewing the Structured Clinical Interview for DSM diagnosis (SCID-IV)^80^ data and clinical information. Total antipsychotic dose and duration were collected. Substance abuse in the previous month or dependence 6 months prior to enrollment, mental retardation per DSM-IV, serious neurological/medical illnesses were exclusion criteria. After explaining the experimental procedures, informed consents were obtained from the subjects. University of Pittsburgh IRB approved the study.

#### Imaging procedures

Details of ^31^P MRS data acquisition and processing are published^11^. Briefly, whole-brain, multi-voxel, in vivo ^31^P MRS data in 3-dimensions was collected on a 3 T Siemens Tim Trio system using a dual-tuned ¹H-³¹P volume head coil and a conventional chemical shift imaging (CSI) sequence. Acquisition parameters were: FOV = 310 × 310 × 160 mm, acquired phase-encoding steps = 14 × 14 × 8 and zero-filled to 16 × 16 × 8 (nominal voxel dimension = 1.94 × 1.94 × 2.0 cm^3^), TR = 0.54 sec, flip-angle = 33^0^ reflecting the Ernst angle where the average T_1_ value of phosphocreatine (PCr), PE, PC was 3 sec, complex data points = 2048, spectral bandwidth = 4.0 kHz, 24 averages of the CSI matrix in which the averaging was weighted to the central k-space points conforming to a 3D elliptical function and pre-acquisition delay of 1.4 ms. T_1_-weighted MPRAGE images were collected and used to guide the extraction of ³¹P MRS signal of hypothesized voxels-of-interest using an innovative procedure^81^. In the *k*-space domain of the ³¹P MRS data, a 75% Hamming window was applied, and modeled in the time domain with 23 Gaussian-damped sinusoids (PE, PC, GPE, and GPC as triplets, Pi, MPL_broad_, PCr and dinucleotides as singlets, and adenosine triphosphate (ATP) (two doublets and a triplet)).

The post-processing and metabolite quantification of extracted ^31^P MRS signal was 100% automated^81^. Due to the *lack of ¹H decoupling*, the quantification of the individual phosphomonoesters (PE and PC), and phosphodiesters (GPE and GPC) were indistinguishable. Therefore, summated measures (PE + PC, GPE + GPC) were obtained. The proportion of gray and white matter, and CSF/extra-cortical space was estimated for each voxel-of-interest using a fully automated procedure^82–84^. Since the CSF concentration of ³¹P metabolites is below the detection limit and the ³¹P metabolites are expressed as a percentage relative to the total signal, the correction for CSF fractions is not applicable and do not confound the ³¹P MRS results.

### SAMPLE 2: AOSZ and age-matched HC

#### Clinical evaluations

Early onset was defined as the first appearance of psychotic symptoms before 18 years of age^85,86^. AOSZ were between 14 and 21 years of age and on stable antipsychotic doses for longer than 1 month. SCID-IV^80^ for adults and K-SADS-PL^87^ for adolescents were administered and reviewed for “consensus” diagnosis as noted above. The exclusion criteria were (a) significant present/past history of medical/neurological illness, e.g., epilepsy, head injury, (b) Obstetric complications, e.g., neonatal asphyxia, (c) Mental retardation per DSM-IV, (d) Hyperbilirubinemia requiring transfusion/phototherapy > 2 days, (e) The Apgar score < 7 at 1 and 5 min after birth, (f) Gestational age < 37 or > 42 weeks, (g) Significant substance use including cigarettes > ½ pack a day, alcohol > 2 drinks/day during pregnancy, and h) Preeclampsia/eclampsia during subject’s pregnancy. These data were collected through mothers of subjects and their obstetric records. After explaining the experimental procedures, informed consents were obtained from adult subjects; subjects below 18 years of age provided the assent and then the consent was obtained from the parents or legal guardians. University of Pittsburgh IRB approved the study.

#### Imaging procedures

For this sample, ^*1*^*H-decoupled* ^31^P MRS data was acquired. Scout images were acquired to prescribe ^31^P MRS voxels. Initial maximization of the B_0_ field homogeneity in the ^1^H mode (shimming) and optimization of the ^31^P reference radio frequency (RF) pulse amplitude for a 33° flip angle was conducted. The acquisition sequence includes a single slab-selective excitation RF pulse followed by phase-encoding pulses to spatially encode in 3D. The axial slab was placed parallel to the AC-PC line covering the whole brain. The scanning parameters: FOV = 310 × 310 × 160 mm, slab thickness = 140 mm, phase-encoding steps = 14 × 14 × 8, zero-filled to 16 × 16 × 8 (nominal voxel dimension = 1.94 × 1.94 × 2.0 cm^3^), TR = 0.54 sec, flip-angle = 33° where the average T1 value of PCr, PE, PC, is 3 sec, complex data points = 2048, spectral bandwidth = 4.0 kHz, 24 averages (weighted-average k-space), which are validated^88^, elliptical k-space sampling. To minimize the signal attenuation due to spin-spin relaxation plus T2* within the pre-acquisition delay time, the rise and fall time parameters and duration of the phase encoding pulses are reduced giving a pre-acquisition delay of 1.4 ms. T1 images were acquired to shift voxels. Post-processing and quantification was similar to the non-decoupled data.

### Genetic assays

Subjects were chosen randomly from the larger YASZ and AOSZ cohorts for genetic assays. Characterization of *C4* variants is challenging due to complex linkage disequilibrium in the HLA region and the complexity of *C4* locus. We used digital droplet PCR (ddPCR)^38^ assay to estimate copy numbers of *C4* structural elements (*C4A, C4B, C4L*, and *C4S*). Briefly, AluI-digested genomic DNA was mixed with primer-probe mix for *C4* and a reference locus (RPP30), and 2 × ddPCR Supermix for Probes (Bio-Rad). The oligonucleotide primers and probes used for assaying copy number of *C4A, C4B, C4L*, and *C4S* were synthesized (IDT tech), and the master mix was emulsified into droplets, using a micro-fluidic droplet generator (Bio-Rad) that was subjected to PCR. After PCR, the fluorescence in each droplet was read using a QX100 droplet reader (Bio-Rad), and the data analyzed using the QuantaSoft software (Bio-Rad) and copy numbers deduced.

To discriminate the compound structural forms of *C4* (AL, AS, BL, BS), we amplified a 5.2 kb product that spans the *C4A/B* by long-range PCR. The diluted 5.2 kb PCR product was further PCR amplified with primers specific for *C4AS* and *C4BS*. The ratio of *C4AS* to *C4BS* was used to determine *C4AS* and *C4BS* copy numbers.

### Statistical analysis

We used Student’s *t* tests to examine differences in age, and *χ*^2^ or Fisher’s exact tests to examine distribution of sex and *C4* GCN between schizophrenia and controls. Since the number of univariate tests (*2 sides* *×* *2 metabolites* *×* *14 regions*) would overwhelm hypothesis testing in this relatively small sample, we used multivariate linear modeling within SPSS 25 controlling for age and sex that would require one model for each group. Multivariate models would also account for correlation among the MPL metabolites in the hypothesized regions. Separate regression models were built for GPC + GPE and PC + PE levels for AOSZ and YASZ by including these metabolite levels for all hypothesized regions, diagnosis, age, sex and education as covariates. Since antipsychotic dose did not significantly contribute to MPL metabolite alterations^11^, we did not covary for medications. To test primary hypothesis on the association of GPC + GPE levels with *C4A* GCNs, we built two separate forward stepwise regression models with Akaike Information Criterion (AIC) for the YASZ and the AOSZ cohorts. Smallest AIC was used to guide model selection. The AIC was used as an additional parameter because it penalizes model complexity, similar to other criterion such as Bayesian Information Criterion. We followed a similar approach to test the secondary hypothesis on the relationship of *C4A* repeats with PC + PE, and to explore the association of *C4B* repeats with GPC + GPE and PC + PE levels.

## Results

### Demographic and clinical characteristics

#### Sample 1: YASZ and age-matched HC

Although the eligibility of subjects to enter the study was 18–44 years, mean age of enrolled YASZ (25.74 ± 8.46 years) did not differ from HC (27.07 ± 7.14 years)(*t* = 0.52, *p* = 0.61) (range 18.24 to 43.98 years for YASZ, and 18.60 to 42.10 years for HC with 3 subjects above 35 years in both groups). Mean duration of illness was 2.37 ± 1.62 years. *C4A* (Fisher’s exact test, *p* = 0.69) and *C4B* (Fisher’s exact test, *p* = 0.23) GCN did not show schizophrenia-HC differences (Table 1). We recapitulated our earlier published results on differences in MPL metabolites in a larger YASZ and HC cohort^11^.

**Table 1:** Demographic and clinical characteristics

	**Young adult-onset schizophrenia (*n*** = 15)	**Healthy controls (*n*** = 22)	**Statistics**	**Adolescent-onset schizophrenia (*n*** = 12)	**Healthy controls (*n*** = 15)	**Statistics**
Age (in years)	25.74 ± 8.46*	27.07 ± 7.14*	*t* = 0.52, *p* = 0.61	19.59 ± 1.52*	19.16 ± 1.32*	*t* = 0.76, *p* = 0.47
Sex						
Male	11	5	Fisher’s exact test, *p* = 0.006	10	7	Fisher’s exact test, *p* = 0.11
Female	4	17		2	8	
*C4AL* repeats						
1 copy	4	4	Fisher’s exact test, *p* = 0.69	1	2	Fisher’s exact test, *p* = 1.00
2 or more copies	11	18		11	13	
*C4BL* repeats						
0 copy	8	15	Fisher’s exact test, *p* = 0.23	7	4	Fisher’s exact test, *p* = 0.27
1 copy	5	2		4	9	
2 or more copies	2	5		1	2	
Duration of illness (in years)	2.37 ± 1.62	–	–	3.99 ± 0.90	–	–

The quality of MRS data was measured as SNR, mean Gaussian linewidths of PCr and Cramer-Rao Lower Bound (CRLB) values. Gaussian linewidth of PCr represents the resolution of spectra, where narrower linewidths indicate higher resolution of spectra, and lower CRLB value is the lower bound on the variance of spectral measurements. Our larger cohort, published previously, did not show case-control differences in the mean SNR and mean Gaussian linewidths of PCr, and CRLB values for the PC + PE and GPC + GPE^11^. This sample that was derived from the larger cohort^11^ showed significant differences for PCr Gaussian linewidth for left caudate, hippocampus, DLPFC, thalamus, and the right ACC, IFC and caudate (all *p* < 0.05). However, the CRLB and the SNR values did not differ between the groups for these regions except for the CRLB of GPC + GPE of the left hippocampus (*p* = 0.028).

#### Sample 2: AOSZ and age-matched HC

Age at onset of psychosis in the AOSZ was 15.60 ± 0.8 years. Age at scan was not significantly different between AOSZ (*n* = 12; 19.59 ± 1.60 years) and HC (*n* = 15; 19.16 ± 1.29 years) (*t* = 0.76, *p* = 0.47). AOSZ were significantly younger than YASZ (*t* = 2.74, d. *f* = 25, p = 0.011) (range 15.33 to 20.92 in both groups). We did not observe AOSZ-HC differences in *C4AL* (Fisher’s exact test, *p* = 1.00) and *C4BL* (Fisher’s exact test, *p* = 0.27) distribution in this sample, as well (Table 1).

Quality of the ^1^H-decoupled ^31^P MRS was high providing improved spectral resolution and peak separation to enable clear separation of PE, PC, GPC, and GPE. The mean SNR of PCr, the mean Gaussian linewidths of the PCr and the CRLB for the PC, PE, GPC, and GPE did not show significant case-control differences except for the CRLB of PC and GPC of the left ventral hippocampus (*p* = 0.025), and the CRLB of PE of the right anterior cingulate (*p* = 0.037). These regions did not show differences associated with *C4* GCN.

### *C4A* variants and MPL metabolites

#### Sample 1: YASZ and age-matched HC

In the combined sample of YASZ and HC, increasing *C4A* GCN were associated with elevated GPC + GPE levels in the ACC while IFC showed a concurrently decreased GPC + GPE and PC + PE levels. The smallest AIC for model selection was −46.20 for GPC + GPE and −43.69 for PC + PE.

**Fig. 1:**
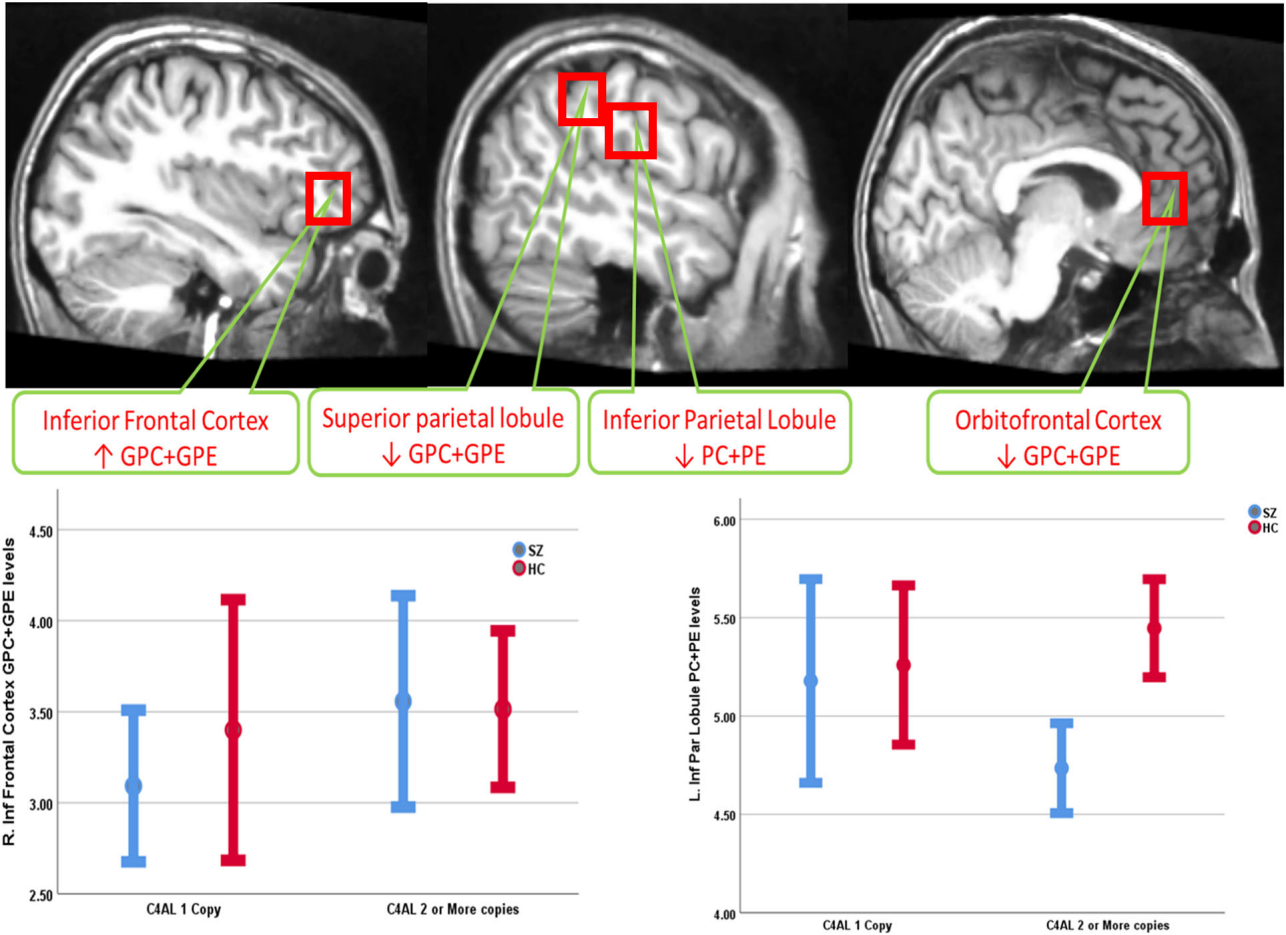
MRS voxels that showed significant MPL metabolite changes among adult-onset schizophrenia (YASZ)

Among YASZ, increasing *C4A* GCN was associated with elevated GPC + GPE levels in a ventral brain region, namely the right IFC with a large effect size (Cohen’s *d* = 1.15) but not among HC. We, also, observed decreased GPC + GPE levels in the ventral regions among YASZ (the left OFC; Cohen’s *d* = 0.85) and HC (left IFC; Cohen’s *d* = 0.52); however, HC showed a concurrent decrease in the left IFC (Cohen’s *d* = 0.57). YASZ patients showed decreased GPC + GPE levels (the right SPL; Cohen’s *d* = 1.8) and decreased PC + PE levels (left IPL; Cohen’s *d* = 0.84) in relation to increasing *C4A* repeats. Overall model AIC was −23.87 for GPC + GPE and −19.72 for PC + PE (Table 2; Fig. 1).

**Table 2:** Association of *C4AL* copy number repeats with MPL metabolite levels among young adult-onset schizophrenia (YASZ) and matched healthy controls

	**GPC** **+** **GPE ↑**	**GPC** **+** **GPE ↓**	**PC** **+** **PE↑**	**PC** **+** **PE↓**
Sample 1: Young adult-onset schizophrenia and age-matched HC				
**YASZ** + **HC**	*L. Anterior Cingulate Cortex* (AIC = −45.70; *β* = 0.45, *t* = 2.62, *p* = 0.013) (*d* = 0.43)	*L. Inferior Frontal Cortex* (AIC = −43.48; *β* = −0.27, *t* = 3.48, *p* = 0.001) (*d* = 0.57)	–	*L. Inferior Frontal Cortex* (AIC = −43.69; *β* = −0.11, *t* = 2.26, *p* = 0.03) (*d* = 0.37)
**YASZ**	*R. Inferior Frontal Cortex* (AIC = −20.73; *β* = 0.37, *t* = 3.81, *p* = 0.007) (*d* *=* 1.15)	*R. Superior Parietal Lobule* (AIC = −15.82; *β* = −0.58, *t* = 5.98, *p* = 0.001) (*d* *=* 1.8*)* *L. Orbitofrontal Cortex* (AIC = −23.87; *β* = −0.15, t = 2.83, p = 0.03) (*d* *=* 0.85)	–	*L. Inferior Parietal Lobule* (AIC = −18.06; β = −0.88, *t* = 3.26, *p* = 0.007) (*d* *=* 0.84)
**HC**	–	*L. Inferior Frontal Cortex* (AIC = −25.90; *β* = −0.17, t = 2.42, p = 0.026) (*d* *=* 0.52)	–	*L. Inferior Frontal Cortex* (AIC = −29.06; *β* = −0.17, *t* = 2.72, *p* = 0.014) (*d* *=* 0.57)
Sample 2: Adolescent-onset schizophrenia and age-matched HC				
**AOSZ** **+** **HC**	*R. Dorsolateral Prefrontal Cortex* (AIC = −44.78; *β* = 0.019, *t* = 2.40, *p* = 0.024) (*d* *=* 0.46)	*L. Anterior Cingulate Cortex* (AIC = −41.49; *β* = −0.088, *t* = 5.05, *p* < 0.001) (*d* *=* 0.99)	–	*R. Thalamus* (AIC = −35.54; *β* = −0.096, *t* = 2.96, *p* = 0.007) (*d* *=* 0.57)

Increased GPC + GPE suggest increased neuropil contraction while decreased PC + PE suggest decreased neuropil expansion

#### Sample 2: AOSZ and age-matched HC

Because there was one AOSZ and two controls with 1 copy of *C4A*, we examined the association of *C4A* repeats in the entire sample of AOSZ and HC. We noted elevated GPC + GPE levels in the DLPFC (Cohen’s *d* = 0.46) but decreasing GPC + GPE levels in the left anterior cingulate with increasing *C4A* GCN.

In the same combined sample, we noted decreasing PC + PE levels in the right thalamus associated with increasing *C4A* GCN (Cohen’s *d* = 0.57). Overall model AIC was −44.78 for GPC + GPE and −36.52 for PC + PE (Table 2; Fig. 1).

### Post hoc tests for the association of *C4BL* repeats with MPL metabolite changes

#### Sample 1: YASZ and age-matched HC

Combined sample of YASZ + HC showed increased GPC + GPE levels in the left IPL with increasing *C4BL* repeats. Right ventral hippocampus showed elevated PC + PE and right DLFPC showed decreased PC + PE. The AIC for overall model selection was −77.07 for GPC + GPE and −18.13 for PC + PE. YASZ subjects showed elevated GPC + GPE levels in the cerebellar vermis and the left STG along with decreased PC + PE levels in the left IPL and OFC with increasing *C4BL* repeats. HC showed increased GPC + GPE levels in multiple regions with decreased PC + PE levels in the DLPFC and caudate. The lowest AIC for model selection was 10.92 for GPC + GPE and −13.89 for PC + PE for within YASZ and HC.

#### Sample 2: AOSZ and age-matched HC

**Fig. 2:**
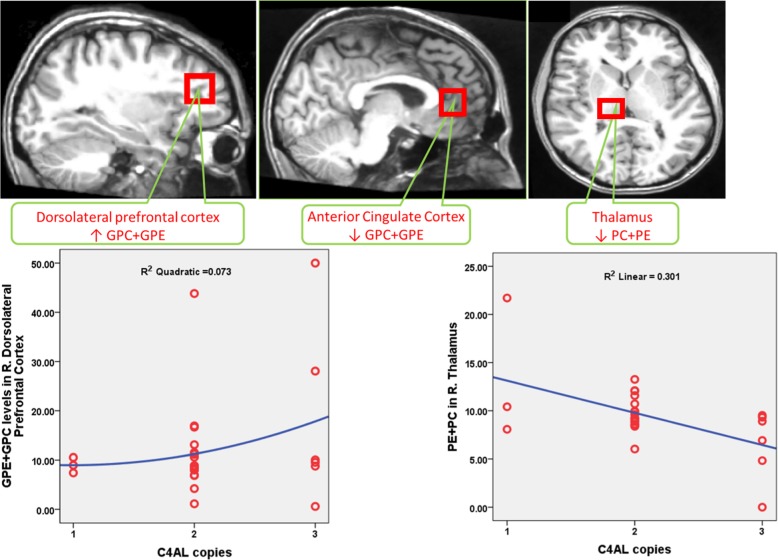
MRS voxels that showed significant MPL metabolite changes among adolescent-onset schizophrenia (AOSZ) in relation to *C4AL* repeats

Because of small n in each cell, AOSZ and HC groups were not examined separately. MPL metabolite changes in relation to *C4B* GCN were observed in the prefrontal and subcortical regions in the combined sample of AOSZ and HC. PC + PE levels did not differ between with *C4BL* repeats (Table 3; Fig. 2). AIC for overall model selection was −53.13 for GPC + GPE.

**Table 3:** Exploratory analysis of association of *C4BL* copy number repeats with MPL metabolite levels among young adult-onset schizophrenia (YASZ) and matched healthy controls, and a combined sample of AOSZ + HC

	**GPC** **+** **GPE↑**	**GPC** **+** **GPE↓**	**PC** **+** **PE↑**	**PC** **+** **PE↓**
Sample 1: Young adult-onset schizophrenia and age-matched HC				
**YASZ** **+** **HC**	*L. Inferior Parietal Lobule* (AIC = −32.32; *β* = 0.32, *t* = 3.43, *p* = 0.002) (*d* = 0.56)	–	*R. Vent Hippocampus* (AIC = −17.92; *β* = 0.25, *t* = 2.31, *p* = 0.027) (*d* = 0.38)	*R. Dorsolateral Prefrontal Cortex* (AIC = −19.50; *β* = −0.38, *t* = 2.65, *p* = 0.012); (*d* = 0.44)
**YASZ**	*Cerebellar Vermis* (AIC = −9.64; *β* = 0.42, *t* = 2.69, *p* = 0.02) (*d* *=* 0.69)	–	*L. Inferior Parietal Lobule* (AIC = −13.44; *β* = 0.91, *t* = 3.37, *p* = 0.006) (*d* *=* 0.87)	–
	*L. Superior Temporal Gyrus* (AIC = −10.89; *β* = 0.48, *t* = 2.60, *p* = 0.025) (*d* *=* 0.67)	–	*L. Orbitofrontal Cortex* (AIC = −10.25; *β* = 0.18, *t* = 2.74, *p* = 0.019) (*d* *=* 0.71)	–
**HC**	*L. Superior Parietal Lobule* (AIC = −18.04; *β* = 1.86, *t* = 37.20, *p* < 0.001) (*d* *=* *7*.93)	*L. Superior Temporal Gyrus* (AIC = −30.15; *β* = −1.05, *t* = 31.78, *p* < 0.001) (*d* *=* 6.77)	*Posterior Cingulate Cortex* (AIC = −12.43; *β* = 0.55, *t* = 3.45, *p* = 0.003) (*d* *=* *0*.73)	*R. Dorsolateral Prefrontal Cortex* (AIC = −10.78; *β* = −0.38, *t* = 3.50, *p* = 0.003) (*d* *=* 0.75)
	*Posterior Cingulate Cortex* (AIC = −15.18; *β* = 1.42, *t* = 31.15, *p* < 0.001) (*d* *=* 6.61)	*L. Dorsolateral Prefrontal Cortex* (AIC = −15.18; *β* = −0.74, *t* = 25.72, *p* < = 0.001) (*d* *=* 5.41)	*L. Hippocampus* (AIC = −18.13; *β* = 0.37, *t* = 2.37, *p* = 0.031) (*d* *=* 0.50)	*R. Caudate* (AIC = −15.36; *β* = −0.71, *t* = 3.40, *p* = 0.004) (*d* *=* 0.73)
	*L. Caudate* (AIC = −41.64; *β* = 0.45, *t* = 12.31, *p* < 0.001) (*d* *=* 2.61)	*L. Dorsal Hippocampus* (AIC = −46.70; *β* = −0.65, *t* = 13.34, *p* < 0.001) (*d* *=* 2.87)		
	*L. Ventral Hippocampus* (AIC = −74.51; *β* = 0.08, *t* = 4.74, *p* = 0.001) (*d* *=* 1.00)	*R. Anterior Cingulate Cortex* (AIC = −37.77; *β* = −0.26, *t* = 12.57, *p* < 0.001) (*d* *=* 2.63)		
Sample 2: Adolescent-onset schizophrenia and age-matched HC				
**AOSZ** **+** **HC**	*R. Caudate* AIC = −34.93; (β = 0.16, *t* = 10.07, *p* < 0.001) (*d* *=* 1.95)	*R. Thalamus* (AIC = −37.35; *β* = −0.14, *t* = 6.68, *p* < 0.001) (*d* *=* 1.26)	–	–
	*R. Hippocampus* (AIC = − 34.92; *β* = 0.16, *t* = 8.07, *p* < 0.001) (*d* *=* 1.56)	*R. Anterior Cingulate Cortex* (AIC = −42.36; *β* = −0.03, *t* = 5.27, *p* < 0.001) (*d* *=* 1.09)	–	–
	*R. Dorsolateral Prefrontal Cortex* (AIC = −49.12; *β* = 0.03, *t* = 6.21, *p* < 0.001) (*d* *=* 1.19)	*L. Dorsolateral Prefrontal Cortex* (AIC = −41.64; *β* = −0.03, *t* = 2.18, *p* = 0.046) (*d* *=* 0.44)	–	–
	*Ventral Prefrontal Cortex* (AIC = −46.07; *β* = 0.04, *t* = 5.50, *p* < 0.001) (*d* *=* 1.03)	–	–	–
	*L. Orbitofrontal Cortex* (AIC = −34.93; *β* = 0.03, *t* = 5.34, *p* < 0.001) (*d* *=* 1.01)	–	–	–
	*L. Hippocampus* (AIC = −34.93; *β* = 0.09, *t* = 2.95, *p* = 0.011) (*d* = 0.56)	–	–	–

Increased GPC + GPE suggest increased neuropil contraction while decreased PC + PE suggest decreased synapse expansion

## Discussion

This is the first study, to our knowledge, to demonstrate the association of *C4A* repeats with increased neuropil contraction as shown by elevated GPC + GPE levels and/or decreased PC + PE levels in two independent cohorts of schizophrenia patients. Neuropil contraction was observed in the prefrontal and parietal regions among adult-onset schizophrenia patients whereas adolescent subjects (AOSZ and controls) showed neuropil contraction in the prefrontal and thalamic regions. Our hypothesis that the GPC + GPE levels would be elevated with increasing *C4A* repeats in the frontal (DLFPC, IFC, VPFC), cingulate (ACC, PCC), parietal (IPL, SPL), the OFC and the cerebellum in both cohorts was partly supported. Our second hypothesis was, also, partly supported for the YASZ cohort in that the YASZ showed elevated GPC + GPE and/or decreased PC + PE levels in a ventral region (IFC) but not all hypothesized regions. Likewise, AOSZ + HC group showed decreased PC + PE levels in the thalamus. Overall, our results on patients and controls support the association of *C4* deficiency with synaptic pruning observed in the rodent model, although the pattern of associations are intriguing. Further, this study extends Sekar et al.^38^ rodent model findings to human subjects with schizophrenia and for *C4B* associations with neuropil contraction although *C4B* was not associated with risk for schizophrenia.

Changes in MPL metabolite levels measured through ^31^P MRS reflects expansion/contraction of membranes that contain the MPLs (phosphatidylcholine, PtdC; phosphatidylethanolamine, PtdE). Expansion/contraction of cell membranes that primarily occurs at the axonal endings and dendritic branches during neuropil formation/contraction are associated with elevated MPL precursors (PE, PC) and catabolites (GPC, GPE), respectively. Changes in myelination, neuronal soma size and glial cells contribute considerably less to MPL metabolite signals on ^31^P MRS^59^. Thus, major source of MPL metabolite signals is likely to be the synaptically dense neuropil^68,69^ (see our prior publication for details^11^). Since postmortem studies have not consistently reported atrophy of neuropil^89^, observed changes in MPL metabolites are unlikely to be due to general neuropil atrophy.

The first in vivo evidence of altered neuropil development in first-episode neuroleptic-naïve schizophrenia was provided by ^31^P MRS data (decreased PC + PE and increased GPC + GPE), later supported by postmortem neuropil morphology data^90–92^. Neuropil reduction may be linked to dendritic spine loss^91,93–95^ and decreased neuronal soma size^96,97^ as observed in postmortem brain tissue of schizophrenia patients. Since dendritic spines receive majority of cortical excitatory synapses, dendritic spine loss may suggest loss of cortical excitatory synapses. Thus, the association of *C4A* repeats with neuropil contraction may support synaptic pruning in the rodent model^38^. However, other factors may also contribute to neuropil reduction, e.g., peripheral blood inflammatory mediators C-reactive protein (CRP) and Interleukin-6 (IL-6) levels^11^ but unlikely to be due to long-term administration of antipsychotics. Association of changes in MPL metabolites with antipsychotic medications has been examined in several studies. These studies show that MPL metabolites were altered with short-term but not long-term treatment^98–101^. Given short duration of illness of both cohorts and lack of association of medications with MPL metabolites, antipsychotics may not have contributed a major variance to MPL metabolite differences in this study. Therefore, genetic influence of a variant that showed genomewide significance on neuropil changes exemplify an attempt to explore in vivo biological significance of such variants.

Among six brain regions that showed increased *C4A* and *C4B* RNA expression associated with *C4* repeats in postmortem brain tissue of schizophrenia patients^38^, we found neuropil alterations associated with *C4A* GCN in four of five regions examined. While the frontal (DLPFC and the IFC) and parietal (IPL) regions showed increased neuropil contraction, the ACC and the OFC showed decreased neuropil contraction without altered neuropil formation. HC showed decreased neuropil turnover in the IFC. Further, the thalamus (that was not examined in the previous postmortem study^38^) showed decreased neuropil formation in the AOSZ + HC cohort. Cerebellum did not show neuropil contraction associated with higher *C4A* GCN. Taking our in vivo ^31^P MRS data with the published data on increased RNA expression in these regions suggests that abnormal function of *C4* GCN contributes to neuropil changes in schizophrenia. However, YASZ showed variations in widespread cortical regions with greater heterogeneity in neuropil contraction/expansion compared to HC. Precise reasons for such heterogeneous associations are unclear. Data on differential expression of *C4A* in various regions of the brain within the context of illness course or neurodevelopment are not available. Examination of whole brain ^31^P MRS data allowed us to identify brain regions beyond those examined in the postmortem study that suggests that *C4AL* may be associated with decreased neuropil synthesis, as well as neuropil contraction, both of which may be observed as dendritic spine loss–a proxy measure of synaptic pruning in postmortem studies. In the combined AOSZ + HC sample, *C4AL* showed neuropil changes in the ventral and subcortical regions. Partial support for both hypothesis may possibly be because our YASZ were not in the third decade and AOSZ were not in early adolescence where the neurodevelopment would be more active such as in early to mid-adolescence. Future postmortem and animal studies should examine these issues.

Furthermore, since the AOSZ cohort was significantly younger than the YASZ, our prediction that the AOSZ and YASZ would show regionally distinct patterns of MPL metabolite changes in relation to *C4* GCNs broadly reflecting neurodevelopmental trajectory was not supported. This may be because the age at scan was about 19 years and the neurodevelopment might have progressed beyond dorsal regions into the ventral regions. Another reason may be inadequate power of our sample size to detect such differences. Further studies are needed to investigate developmental effects of *C4A* and *C4B* repeats.

We did not observe increased neuropil contraction among young adult HC although a concurrent reduction in PC + PE and GPC + GPE was observed in relation to *C4AL*. Exploratory analysis on the association of *C4BL* with MPL metabolites showed significant associations with increased neuropil contraction among young-adult HC compared to YASZ patients. Precise reasons are unclear; however, it is possible that the pattern of expression of *C4A* and *C4B* proteins may be different in patients compared to healthy subjects.

This study supported our secondary hypothesis of decreased neuropil expansion. It can be postulated that *C4* GCN may affect each brain region through increased neuropil contraction or decreased formation. Although postmortem examination allows direct examination of spines, differentiation of decreased spine formation from increased pruning by cross-sectional examination is challenging. Unique advantage of in vivo MRS data is its ability to distinguish between increased contraction and diminished formation to the neuropil density. Thus, the findings of this study, if replicated, raises the possibility of the contribution of *C4* GCN to decreased neuropil formation for future cellular and animal studies.

*C4BL* GCN were associated with MPL metabolite changes in the dorsal and subcortical regions among adult-onset schizophrenia patients. However, controls showed a similar pattern but in a distinct set of regions and were more extensive compared to patients. The associations were noted in the ventral and subcortical regions among adolescent cohort contrary to our predictions. One likely reason is that nearly half of subjects in both groups did not have any copies of *C4BL*, effectively making it a comparison between subjects with and without *C4BL* GCN. Further studies with larger samples are required.

The quality of the spectral data acquired with (AOSZ) and without (YASZ) ^1^H-decoupling was reasonably good, although the ^1^H-decoupled MRS data was expectedly of higher quality. The SNR and CRLB values of ^31^P MRS data without decoupling did not show significant differences but the PCr Gaussian linewidth was longer for selected voxels suggests that the variability among the groups was within reasonable limits. The higher quality of ^1^H-decoupled data suggests that higher strength magnets may be able to offer better peak separation and quantification of spectral components.

The strengths of our study include examination of two independent schizophrenia cohorts and HC groups matched for each group that shows consistency of association of *C4A* repeats with neuropil contraction, and extends the findings to decreased neuropil formation. Examining ^31^P MRS data acquired with and without ^1^H-decoupling show predicted associations with neuropil contraction supporting robustness of the findings. Innovative ddPCR assays with excellent quality control adds to these strengths. Demonstrating the associations of *C4* GCN within the human context is important because *C4A* is human-specific and supplements the rodent model observations. Examining early course patients minimizes the confounds of prolonged medication exposure, illness chronicity, and comorbid illnesses and their treatment that may affect postmortem data. Limitations of our study include relatively small sample sizes. Temporal changes could not be inferred in this cross-sectional study. ^31^P MRS data suggests changes in the neuropil that is a synaptically dense region with few cell bodies but does not directly measure changes in synapses, dendritic arborization or spine density.

In summary, our study reports association of increased contraction and decreased formation of neuropil with the GCN of *C4* variants. Although data from gene expression studies on postmortem brain tissues and animal models are persuasive, corroboration with live human patients is critical to advance the knowledge of biological significance of gene variants observed in repeatedly replicated GWA studies. Although *C4B* was not associated with schizophrenia risk in Sekar et al. study, it is likely that *C4B* may contribute to neuropil alterations that requires further studies. Larger sample sizes need to be examined to replicate our findings.
